# 5′′-(4-Chloro­benzyl­idene)-1′-(4-chloro­phen­yl)-1′′-methyl-1′,2′,3′,5′,6′,7′,8′,8a’-octa­hydro­dispiro­[acenaphthyl­ene-1,3′-indolizine-2′,3′′-piperidine]-2,4′′(1*H*)-dione

**DOI:** 10.1107/S160053681204545X

**Published:** 2012-11-10

**Authors:** J. Suresh, R. A. Nagalakshmi, R. Ranjith Kumar, S. Sivakumar, P. L. Nilantha Lakshman

**Affiliations:** a Department of Physics, The Madura College, Madurai 625 011, India; b Department of Organic Chemistry, School of Chemistry, Madurai Kamaraj University, Madurai 625 021, India; c Department of Food Science and Technology, University of Ruhuna, Mapalana, Kamburupitiya 81100, Sri Lanka

## Abstract

In the title compound, C_37_H_32_Cl_2_N_2_O_2_, the pyridinone ring adopts a twisted half-chair conformation. The fused pyrrolidine and piperidine rings of the octa­hydro­indolizine unit exhibit envelope (with the C atom bound to the C atom bearing the chloro­benzene ring being the flap atom) and chair conformations, respectively. The dihedral angle between the chloro­benzene rings is 84.03 (1)°. In the crystal, C—H⋯π inter­actions lead to supra­molecular chains along [101] that assemble in the *ac* plane. Connections along the *b* axis are of the type Cl⋯Cl [3.4065 (8) Å].

## Related literature

For general properties of indolizines, see: Gundersen *et al.* (2007 ▶). For related structures, see: Sussman & Wodak (1973 ▶); Wodak (1975 ▶). For ring conformation analysis, see: Cremer & Pople (1975 ▶).
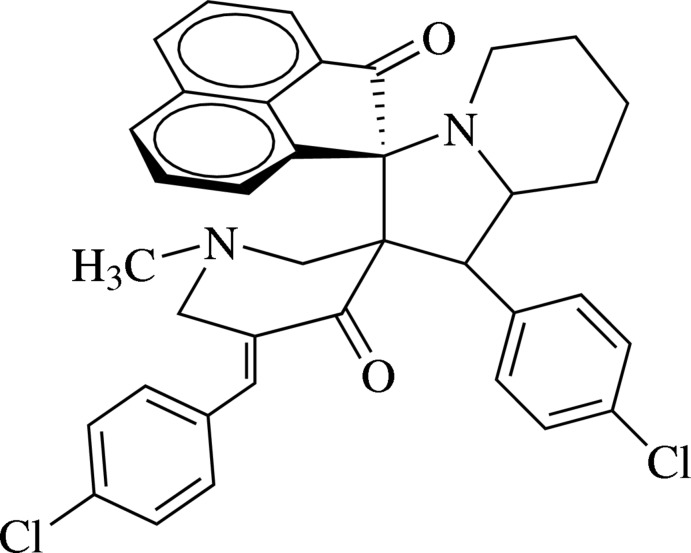


## Experimental

### 

#### Crystal data

C_37_H_32_Cl_2_N_2_O_2_
*M* *_r_* = 607.55
Monoclinic, 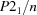

*a* = 14.1346 (5) Å
*b* = 15.2184 (6) Å
*c* = 14.5603 (6) Å
β = 102.337 (1)°
*V* = 3059.7 (2) Å^3^
*Z* = 4
Mo *K*α radiation
μ = 0.25 mm^−1^
*T* = 293 K
0.21 × 0.19 × 0.18 mm

#### Data collection

Bruker Kappa APEXII diffractometer
Absorption correction: multi-scan (*SADABS*; Sheldrick, 1996 ▶) *T*_min_ = 0.967, *T*_max_ = 0.974
32144 measured reflections
7321 independent reflections
4492 reflections with *I* > 2σ(*I*)
*R*_int_ = 0.041

#### Refinement

*R*[*F* ^2^ > 2σ(*F* ^2^)] = 0.046
*wR*(*F* ^2^) = 0.113
*S* = 1.01
7321 reflections
389 parameters
H-atom parameters constrained
Δρ_max_ = 0.23 e Å^−3^
Δρ_min_ = −0.38 e Å^−3^

### 

Data collection: *APEX2* (Bruker, 2004 ▶); cell refinement: *SAINT* (Bruker, 2004 ▶); data reduction: *SAINT*; program(s) used to solve structure: *SHELXS97* (Sheldrick, 2008 ▶); program(s) used to refine structure: *SHELXL97* (Sheldrick, 2008 ▶); molecular graphics: *PLATON* (Spek, 2009 ▶); software used to prepare material for publication: *SHELXL97*.

## Supplementary Material

Crystal structure: contains datablock(s) global, I. DOI: 10.1107/S160053681204545X/tk5167sup1.cif

Structure factors: contains datablock(s) I. DOI: 10.1107/S160053681204545X/tk5167Isup2.hkl

Additional supplementary materials:  crystallographic information; 3D view; checkCIF report

## Figures and Tables

**Table 1:** Hydrogen-bond geometry (Å, °) *Cg*1 is the centroid of the C52–C57 benzene ring.

*D*—H⋯*A*	*D*—H	H⋯*A*	*D*⋯*A*	*D*—H⋯*A*
C75—H75⋯*Cg*1^i^	0.93	2.88	3.669 (2)	144

Symmetry code: (i) 
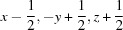
.

**Fig. 1.**
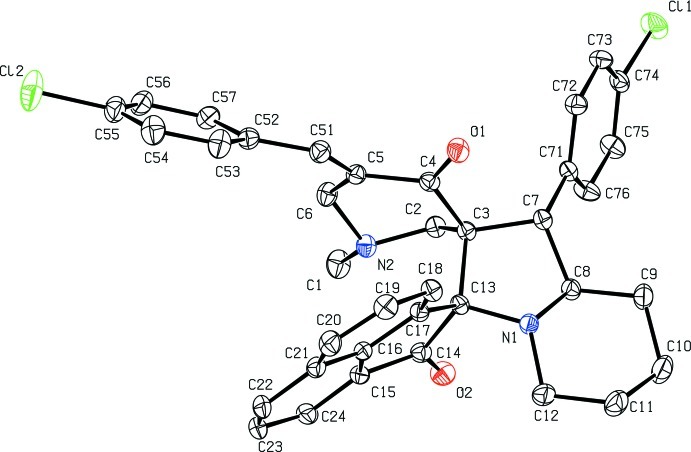
The molecular structure of (I), showing 50% probability displacement ellipsoids and the atom-numbering scheme. Hydrogen atoms have been omitted.

## supplementary crystallographic information

### Comment

Indolizines are electron-rich heterocycles with very low oxidation potentials. Functionalised indolizines are common substructures found in biologically important natural products and synthetic pharmaceuticals. Due to the various biological functions associated with this skeleton, it has been frequently employed as a key scaffold in the drug industry (Gundersen *et al.*, 2007).

In the title compound (Fig.1), the pyridinone ring adopts twisted half chair conformation with atoms N2 and C3 deviating by 0.6030 (1) Å and 0.4814 (1) Å respectively from the least squares planes defined by other atoms (C2/C4/C5/C6). Within the octahydroindolizine, the six membered piperidine ring adopts a chair conformation as evident from the puckering parameters Q = 0.567 (2) Å, θ = 180 (2)° and Φ = 50 (8)° (Cremer & Pople, 1975). The dihedral angle between the two chlorobenzene rings is 84.03 (1)°, and these rings (C71—C76) and (C52—C57) form angles of 80.91 (1) and 32.50 (1)°, respectively, with the plane defined by atoms (C2/C4/C5/C6) of pyridinone ring. Each of the carbonyl bond lengths, *i.e* C4=O1 and C14=O2, is 1.214 (2) Å, and each of these atoms participates in two intramolecular C—H···O contacts, with the closest of these listed in Table 1. The C8—N1 bond length (1.454 (2) Å) is comparable with the C*sp*^2^—N*sp*^2^ distance found in the similar structures (Sussman & Wodak, 1973; Wodak, 1975).

A weak intermolecular C—H···π interaction, *viz*. C75—H75···*Cg*1 (*Cg*1 is the centroid of the ring C52—C57; symmetry code is given in Table 1) is observed. These lead to supramolecular chains along [101] that assemble in the *ac* plane. Connections between layers are of the type Cl···Cl (Cl1···Cl1^i^ = 3.4065 (8) Å: symmetry code: 1 - *x*, 1 - *y*, 2 - *z*).

### Experimental

A mixture of 1-methyl-3,5-bis[(*E*)-4-cholromethylidene]tetrahydro-4(1*H*)- pyridinone (1 mmol), acenaphthenequinone (1 mmol) and piperidine-2- carboxylic acid (1 mmol) was dissolved in isopropyl alcohol (15 ml) and heated to reflux for 60 min. After completion of the reaction, as evident from TLC, the mixture was poured into water (50 ml), the precipitated solid was filtered and washed with water (100 ml) to obtain pure yellow solid. Melting point: 518 K, Yield: 93%

### Refinement

H atoms were placed at calculated positions and allowed to ride on their carrier atoms with C—H = 0.93–0.98 Å; *U*_iso_ = 1.2*U*_eq_(C) for CH_2_ and CH groups, and *U*_iso_ = 1.5*U*_eq_(C) for CH_3_ groups. The (-1 0 1) reflection was probably affected by the beam-stop and was omitted from the refinement.

### Crystal data

**Table:**

C_37_H_32_Cl_2_N_2_O_2_	*F*(000) = 1272
*M**_r_* = 607.55	*D*_x_ = 1.319 Mg m^−^^3^
Monoclinic, *P*2_1_/*n*	Mo *K*α radiation, λ = 0.71073 Å
Hall symbol: -P 2yn	Cell parameters from 2000 reflections
*a* = 14.1346 (5) Å	θ = 2–31°
*b* = 15.2184 (6) Å	µ = 0.25 mm^−^^1^
*c* = 14.5603 (6) Å	*T* = 293 K
β = 102.337 (1)°	Block, yellow
*V* = 3059.7 (2) Å^3^	0.21 × 0.19 × 0.18 mm
*Z* = 4	

### Data collection

**Table:**

Bruker Kappa APEXII diffractometer	7321 independent reflections
Radiation source: fine-focus sealed tube	4492 reflections with *I* > 2σ(*I*)
Graphite monochromator	*R*_int_ = 0.041
Detector resolution: 0 pixels mm^-1^	θ_max_ = 27.9°, θ_min_ = 2.0°
ω and φ scans	*h* = −18→16
Absorption correction: multi-scan (*SADABS*; Sheldrick, 1996)	*k* = −16→20
*T*_min_ = 0.967, *T*_max_ = 0.974	*l* = −19→17
32144 measured reflections	

### Refinement

**Table:**

Refinement on *F*^2^	Secondary atom site location: difference Fourier map
Least-squares matrix: full	Hydrogen site location: inferred from neighbouring sites
*R*[*F*^2^ > 2σ(*F*^2^)] = 0.046	H-atom parameters constrained
*wR*(*F*^2^) = 0.113	*w* = 1/[σ^2^(*F*_o_^2^) + (0.0403*P*)^2^ + 0.7517*P*] where *P* = (*F*_o_^2^ + 2*F*_c_^2^)/3
*S* = 1.01	(Δ/σ)_max_ < 0.001
7321 reflections	Δρ_max_ = 0.23 e Å^−^^3^
389 parameters	Δρ_min_ = −0.38 e Å^−^^3^
0 restraints	Extinction correction: *SHELXL97* (Sheldrick, 2008), Fc^*^=kFc[1+0.001xFc^2^λ^3^/sin(2θ)]^-1/4^
Primary atom site location: structure-invariant direct methods	Extinction coefficient: 0.0023 (4)

### Special details

**Table:**

Geometry. All e.s.d.'s (except the e.s.d. in the dihedral angle between two l.s. planes) are estimated using the full covariance matrix. The cell e.s.d.'s are taken into account individually in the estimation of e.s.d.'s in distances, angles and torsion angles; correlations between e.s.d.'s in cell parameters are only used when they are defined by crystal symmetry. An approximate (isotropic) treatment of cell e.s.d.'s is used for estimating e.s.d.'s involving l.s. planes.
Refinement. Refinement of *F*^2^ against ALL reflections. The weighted *R*-factor *wR* and goodness of fit *S* are based on *F*^2^, conventional *R*-factors *R* are based on *F*, with *F* set to zero for negative *F*^2^. The threshold expression of *F*^2^ > σ(*F*^2^) is used only for calculating *R*-factors(gt) *etc*. and is not relevant to the choice of reflections for refinement. *R*-factors based on *F*^2^ are statistically about twice as large as those based on *F*, and *R*- factors based on ALL data will be even larger.

### Fractional atomic coordinates and isotropic or equivalent isotropic displacement parameters (Å^2^)

**Table:**

	*x*	*y*	*z*	*U*_iso_*/*U*_eq_	
Cl1	0.04645 (4)	0.07200 (4)	0.43166 (3)	0.07029 (19)	
Cl2	0.71789 (5)	0.05345 (6)	−0.16446 (6)	0.1089 (3)	
O1	0.13905 (9)	0.07134 (8)	−0.05296 (10)	0.0538 (4)	
N1	−0.00938 (10)	0.28748 (10)	−0.08843 (9)	0.0402 (4)	
O2	0.13090 (10)	0.42902 (8)	−0.00055 (9)	0.0547 (4)	
C16	0.18467 (12)	0.32304 (11)	−0.19504 (12)	0.0380 (4)	
C7	0.03322 (12)	0.19119 (11)	0.03419 (11)	0.0368 (4)	
H7	0.0063	0.1416	−0.0060	0.044*	
C13	0.09576 (12)	0.28443 (11)	−0.07824 (11)	0.0356 (4)	
N2	0.28362 (10)	0.29147 (10)	0.05353 (10)	0.0421 (4)	
C4	0.18149 (13)	0.13902 (12)	−0.02618 (11)	0.0383 (4)	
C6	0.34414 (13)	0.21861 (13)	0.03624 (14)	0.0487 (5)	
H6A	0.3748	0.1921	0.0958	0.058*	
H6B	0.3948	0.2407	0.0068	0.058*	
C71	0.04004 (12)	0.16261 (11)	0.13512 (11)	0.0371 (4)	
C51	0.32365 (14)	0.09841 (12)	−0.08389 (13)	0.0473 (5)	
H51	0.2793	0.0603	−0.1200	0.057*	
C21	0.22039 (13)	0.31929 (13)	−0.27798 (13)	0.0463 (5)	
C3	0.12964 (12)	0.21704 (11)	0.00698 (11)	0.0349 (4)	
C17	0.12068 (12)	0.26047 (11)	−0.17163 (11)	0.0363 (4)	
C14	0.14749 (13)	0.37636 (12)	−0.05770 (12)	0.0413 (4)	
C74	0.04679 (13)	0.10710 (13)	0.31784 (12)	0.0453 (5)	
C8	−0.03173 (12)	0.26980 (12)	0.00271 (12)	0.0411 (4)	
H8	−0.0113	0.3195	0.0451	0.049*	
C15	0.20574 (12)	0.39111 (12)	−0.12968 (12)	0.0407 (4)	
C72	0.05102 (14)	0.07488 (12)	0.15926 (13)	0.0458 (5)	
H72	0.0557	0.0337	0.1132	0.055*	
C2	0.20079 (12)	0.26003 (12)	0.08805 (12)	0.0406 (4)	
H2A	0.1698	0.3087	0.1130	0.049*	
H2B	0.2216	0.2177	0.1381	0.049*	
C5	0.28707 (13)	0.14998 (11)	−0.02579 (12)	0.0397 (4)	
C52	0.42240 (14)	0.09202 (12)	−0.10001 (14)	0.0476 (5)	
C19	0.12123 (15)	0.19024 (14)	−0.31828 (13)	0.0529 (5)	
H19	0.0987	0.1454	−0.3607	0.064*	
C24	0.26740 (14)	0.45710 (13)	−0.14340 (15)	0.0506 (5)	
H24	0.2822	0.5033	−0.1010	0.061*	
C76	0.03301 (15)	0.22134 (13)	0.20577 (12)	0.0515 (5)	
H76	0.0262	0.2809	0.1915	0.062*	
C20	0.18534 (15)	0.24935 (14)	−0.33970 (14)	0.0552 (5)	
H20	0.2064	0.2437	−0.3957	0.066*	
C23	0.30756 (14)	0.45261 (15)	−0.22372 (17)	0.0592 (6)	
H23	0.3514	0.4958	−0.2325	0.071*	
C12	−0.06134 (14)	0.36225 (14)	−0.13800 (14)	0.0563 (5)	
H12A	−0.0443	0.3692	−0.1987	0.068*	
H12B	−0.0434	0.4156	−0.1020	0.068*	
C18	0.08774 (13)	0.19465 (12)	−0.23358 (12)	0.0439 (4)	
H18	0.0440	0.1533	−0.2204	0.053*	
C73	0.05515 (14)	0.04700 (13)	0.25014 (13)	0.0502 (5)	
H73	0.0636	−0.0123	0.2653	0.060*	
C75	0.03572 (15)	0.19436 (13)	0.29690 (13)	0.0531 (5)	
H75	0.0301	0.2350	0.3431	0.064*	
C55	0.60436 (16)	0.06903 (14)	−0.13949 (18)	0.0621 (6)	
C54	0.52339 (17)	0.05391 (14)	−0.20863 (17)	0.0667 (6)	
H54	0.5292	0.0367	−0.2684	0.080*	
C9	−0.13872 (14)	0.25407 (15)	−0.00761 (15)	0.0601 (6)	
H9A	−0.1533	0.2466	0.0541	0.072*	
H9B	−0.1569	0.2005	−0.0431	0.072*	
C22	0.28499 (14)	0.38774 (15)	−0.28913 (15)	0.0565 (6)	
H22	0.3123	0.3884	−0.3418	0.068*	
C1	0.33930 (15)	0.35679 (14)	0.11492 (15)	0.0615 (6)	
H1A	0.3930	0.3756	0.0891	0.092*	
H1B	0.3629	0.3317	0.1760	0.092*	
H1C	0.2988	0.4063	0.1203	0.092*	
C53	0.43343 (16)	0.06461 (14)	−0.18817 (16)	0.0615 (6)	
H53	0.3785	0.0532	−0.2346	0.074*	
C57	0.50604 (15)	0.10706 (13)	−0.03210 (14)	0.0534 (5)	
H57	0.5010	0.1252	0.0277	0.064*	
C10	−0.19697 (16)	0.33041 (18)	−0.05758 (16)	0.0737 (7)	
H10A	−0.1842	0.3828	−0.0190	0.088*	
H10B	−0.2656	0.3173	−0.0675	0.088*	
C56	0.59666 (15)	0.09545 (14)	−0.05184 (16)	0.0586 (5)	
H56	0.6521	0.1056	−0.0056	0.070*	
C11	−0.16993 (15)	0.34676 (18)	−0.15152 (15)	0.0703 (7)	
H11A	−0.2047	0.3977	−0.1814	0.084*	
H11B	−0.1885	0.2965	−0.1923	0.084*	

### Atomic displacement parameters (Å^2^)

**Table:**

	*U*^11^	*U*^22^	*U*^33^	*U*^12^	*U*^13^	*U*^23^
Cl1	0.0784 (4)	0.0908 (4)	0.0412 (3)	−0.0074 (3)	0.0117 (3)	0.0230 (3)
Cl2	0.0596 (4)	0.1353 (7)	0.1435 (7)	−0.0031 (4)	0.0477 (4)	−0.0432 (6)
O1	0.0486 (8)	0.0433 (8)	0.0701 (9)	−0.0097 (7)	0.0140 (7)	−0.0120 (7)
N1	0.0354 (8)	0.0510 (9)	0.0346 (7)	0.0028 (7)	0.0082 (6)	0.0081 (7)
O2	0.0640 (9)	0.0452 (8)	0.0559 (8)	−0.0004 (7)	0.0152 (7)	−0.0112 (7)
C16	0.0324 (9)	0.0422 (10)	0.0396 (9)	0.0029 (8)	0.0079 (7)	0.0080 (8)
C7	0.0370 (9)	0.0413 (10)	0.0322 (8)	−0.0053 (8)	0.0074 (7)	−0.0008 (7)
C13	0.0367 (9)	0.0390 (10)	0.0316 (8)	−0.0041 (8)	0.0081 (7)	0.0004 (7)
N2	0.0378 (8)	0.0436 (9)	0.0435 (8)	−0.0079 (7)	0.0058 (7)	−0.0082 (7)
C4	0.0404 (10)	0.0388 (10)	0.0343 (9)	−0.0014 (9)	0.0048 (8)	0.0023 (8)
C6	0.0378 (10)	0.0545 (12)	0.0521 (11)	−0.0036 (9)	0.0061 (8)	−0.0059 (10)
C71	0.0362 (9)	0.0411 (10)	0.0346 (9)	−0.0059 (8)	0.0086 (7)	0.0028 (8)
C51	0.0434 (11)	0.0448 (11)	0.0533 (11)	−0.0020 (9)	0.0091 (9)	−0.0037 (9)
C21	0.0392 (10)	0.0571 (12)	0.0450 (10)	0.0081 (9)	0.0148 (8)	0.0119 (9)
C3	0.0358 (9)	0.0383 (9)	0.0300 (8)	−0.0033 (8)	0.0060 (7)	0.0007 (7)
C17	0.0356 (9)	0.0415 (10)	0.0316 (8)	−0.0004 (8)	0.0068 (7)	0.0029 (8)
C14	0.0420 (10)	0.0400 (10)	0.0402 (9)	0.0005 (9)	0.0052 (8)	0.0009 (8)
C74	0.0402 (10)	0.0591 (12)	0.0360 (9)	−0.0073 (9)	0.0066 (8)	0.0123 (9)
C8	0.0396 (10)	0.0507 (11)	0.0339 (9)	0.0000 (9)	0.0101 (8)	0.0047 (8)
C15	0.0363 (10)	0.0388 (10)	0.0458 (10)	−0.0006 (8)	0.0060 (8)	0.0079 (8)
C72	0.0511 (11)	0.0423 (11)	0.0446 (10)	0.0017 (9)	0.0116 (9)	0.0008 (9)
C2	0.0392 (10)	0.0473 (11)	0.0341 (9)	−0.0033 (9)	0.0051 (7)	−0.0004 (8)
C5	0.0371 (10)	0.0389 (10)	0.0419 (9)	−0.0004 (8)	0.0055 (8)	0.0015 (8)
C52	0.0468 (11)	0.0393 (10)	0.0583 (12)	0.0012 (9)	0.0149 (9)	−0.0043 (9)
C19	0.0588 (13)	0.0590 (13)	0.0412 (10)	0.0016 (11)	0.0111 (9)	−0.0087 (9)
C24	0.0432 (11)	0.0438 (11)	0.0622 (12)	−0.0055 (9)	0.0053 (10)	0.0086 (10)
C76	0.0779 (15)	0.0398 (11)	0.0392 (10)	−0.0039 (10)	0.0176 (10)	0.0036 (8)
C20	0.0577 (13)	0.0709 (14)	0.0423 (11)	0.0082 (11)	0.0229 (10)	−0.0013 (10)
C23	0.0380 (11)	0.0579 (13)	0.0840 (16)	−0.0038 (10)	0.0184 (11)	0.0239 (12)
C12	0.0519 (12)	0.0691 (14)	0.0478 (11)	0.0115 (11)	0.0100 (9)	0.0183 (10)
C18	0.0462 (11)	0.0493 (11)	0.0358 (9)	−0.0052 (9)	0.0080 (8)	−0.0011 (8)
C73	0.0538 (12)	0.0428 (11)	0.0526 (11)	0.0027 (10)	0.0079 (9)	0.0149 (9)
C75	0.0709 (14)	0.0540 (12)	0.0365 (10)	−0.0066 (11)	0.0159 (10)	−0.0019 (9)
C55	0.0493 (13)	0.0520 (13)	0.0901 (17)	0.0005 (11)	0.0260 (12)	−0.0113 (12)
C54	0.0667 (15)	0.0624 (14)	0.0772 (15)	−0.0058 (12)	0.0289 (13)	−0.0253 (12)
C9	0.0394 (11)	0.0851 (16)	0.0581 (12)	0.0032 (11)	0.0157 (9)	0.0171 (12)
C22	0.0424 (11)	0.0698 (14)	0.0638 (13)	0.0049 (11)	0.0259 (10)	0.0193 (12)
C1	0.0508 (12)	0.0633 (14)	0.0671 (13)	−0.0151 (11)	0.0050 (10)	−0.0207 (11)
C53	0.0512 (13)	0.0642 (14)	0.0698 (14)	−0.0068 (11)	0.0146 (11)	−0.0224 (11)
C57	0.0506 (12)	0.0555 (13)	0.0539 (12)	0.0084 (10)	0.0104 (10)	0.0011 (10)
C10	0.0420 (12)	0.109 (2)	0.0717 (15)	0.0186 (13)	0.0155 (11)	0.0181 (14)
C56	0.0438 (12)	0.0599 (13)	0.0708 (14)	0.0061 (10)	0.0093 (10)	−0.0020 (11)
C11	0.0470 (13)	0.0984 (18)	0.0625 (13)	0.0161 (13)	0.0048 (10)	0.0231 (13)

### Geometric parameters (Å, º)

**Table:**

Cl1—C74	1.7422 (17)	C72—H72	0.9300
Cl2—C55	1.736 (2)	C2—H2A	0.9700
O1—C4	1.214 (2)	C2—H2B	0.9700
N1—C8	1.454 (2)	C52—C57	1.389 (3)
N1—C12	1.459 (2)	C52—C53	1.390 (3)
N1—C13	1.462 (2)	C19—C20	1.360 (3)
O2—C14	1.214 (2)	C19—C18	1.414 (2)
C16—C15	1.395 (2)	C19—H19	0.9300
C16—C17	1.405 (2)	C24—C23	1.407 (3)
C16—C21	1.406 (2)	C24—H24	0.9300
C7—C71	1.516 (2)	C76—C75	1.382 (2)
C7—C8	1.518 (2)	C76—H76	0.9300
C7—C3	1.549 (2)	C20—H20	0.9300
C7—H7	0.9800	C23—C22	1.361 (3)
C13—C17	1.520 (2)	C23—H23	0.9300
C13—C14	1.577 (2)	C12—C11	1.524 (3)
C13—C3	1.602 (2)	C12—H12A	0.9700
N2—C2	1.450 (2)	C12—H12B	0.9700
N2—C1	1.451 (2)	C18—H18	0.9300
N2—C6	1.455 (2)	C73—H73	0.9300
C4—C5	1.500 (2)	C75—H75	0.9300
C4—C3	1.527 (2)	C55—C56	1.364 (3)
C6—C5	1.499 (2)	C55—C54	1.373 (3)
C6—H6A	0.9700	C54—C53	1.376 (3)
C6—H6B	0.9700	C54—H54	0.9300
C71—C72	1.381 (2)	C9—C10	1.517 (3)
C71—C76	1.382 (2)	C9—H9A	0.9700
C51—C5	1.336 (2)	C9—H9B	0.9700
C51—C52	1.467 (3)	C22—H22	0.9300
C51—H51	0.9300	C1—H1A	0.9600
C21—C20	1.413 (3)	C1—H1B	0.9600
C21—C22	1.417 (3)	C1—H1C	0.9600
C3—C2	1.525 (2)	C53—H53	0.9300
C17—C18	1.362 (2)	C57—C56	1.383 (3)
C14—C15	1.482 (2)	C57—H57	0.9300
C74—C75	1.364 (3)	C10—C11	1.517 (3)
C74—C73	1.368 (3)	C10—H10A	0.9700
C8—C9	1.507 (3)	C10—H10B	0.9700
C8—H8	0.9800	C56—H56	0.9300
C15—C24	1.372 (2)	C11—H11A	0.9700
C72—C73	1.379 (2)	C11—H11B	0.9700
			
C8—N1—C12	114.29 (14)	C57—C52—C53	117.44 (18)
C8—N1—C13	108.41 (13)	C57—C52—C51	124.71 (18)
C12—N1—C13	117.92 (14)	C53—C52—C51	117.77 (18)
C15—C16—C17	113.11 (15)	C20—C19—C18	122.18 (19)
C15—C16—C21	123.54 (16)	C20—C19—H19	118.9
C17—C16—C21	123.29 (17)	C18—C19—H19	118.9
C71—C7—C8	115.34 (14)	C15—C24—C23	117.86 (19)
C71—C7—C3	116.33 (13)	C15—C24—H24	121.1
C8—C7—C3	103.35 (13)	C23—C24—H24	121.1
C71—C7—H7	107.1	C75—C76—C71	122.07 (18)
C8—C7—H7	107.1	C75—C76—H76	119.0
C3—C7—H7	107.1	C71—C76—H76	119.0
N1—C13—C17	109.97 (13)	C19—C20—C21	120.86 (17)
N1—C13—C14	114.26 (14)	C19—C20—H20	119.6
C17—C13—C14	101.29 (13)	C21—C20—H20	119.6
N1—C13—C3	103.18 (12)	C22—C23—C24	122.75 (18)
C17—C13—C3	116.83 (13)	C22—C23—H23	118.6
C14—C13—C3	111.79 (13)	C24—C23—H23	118.6
C2—N2—C1	112.59 (14)	N1—C12—C11	109.27 (17)
C2—N2—C6	110.89 (14)	N1—C12—H12A	109.8
C1—N2—C6	111.54 (15)	C11—C12—H12A	109.8
O1—C4—C5	121.23 (16)	N1—C12—H12B	109.8
O1—C4—C3	121.60 (16)	C11—C12—H12B	109.8
C5—C4—C3	117.17 (15)	H12A—C12—H12B	108.3
N2—C6—C5	112.01 (15)	C17—C18—C19	118.81 (17)
N2—C6—H6A	109.2	C17—C18—H18	120.6
C5—C6—H6A	109.2	C19—C18—H18	120.6
N2—C6—H6B	109.2	C74—C73—C72	119.55 (17)
C5—C6—H6B	109.2	C74—C73—H73	120.2
H6A—C6—H6B	107.9	C72—C73—H73	120.2
C72—C71—C76	117.25 (16)	C74—C75—C76	118.84 (18)
C72—C71—C7	120.31 (16)	C74—C75—H75	120.6
C76—C71—C7	122.42 (16)	C76—C75—H75	120.6
C5—C51—C52	130.55 (18)	C56—C55—C54	121.0 (2)
C5—C51—H51	114.7	C56—C55—Cl2	119.89 (18)
C52—C51—H51	114.7	C54—C55—Cl2	119.13 (18)
C16—C21—C20	115.82 (17)	C55—C54—C53	119.1 (2)
C16—C21—C22	115.27 (18)	C55—C54—H54	120.5
C20—C21—C22	128.90 (18)	C53—C54—H54	120.5
C2—C3—C4	107.26 (14)	C8—C9—C10	110.89 (17)
C2—C3—C7	112.44 (13)	C8—C9—H9A	109.5
C4—C3—C7	113.04 (14)	C10—C9—H9A	109.5
C2—C3—C13	111.31 (13)	C8—C9—H9B	109.5
C4—C3—C13	109.70 (13)	C10—C9—H9B	109.5
C7—C3—C13	103.12 (13)	H9A—C9—H9B	108.0
C18—C17—C16	119.00 (16)	C23—C22—C21	120.88 (19)
C18—C17—C13	131.08 (15)	C23—C22—H22	119.6
C16—C17—C13	109.81 (14)	C21—C22—H22	119.6
O2—C14—C15	127.07 (17)	N2—C1—H1A	109.5
O2—C14—C13	124.48 (16)	N2—C1—H1B	109.5
C15—C14—C13	107.78 (14)	H1A—C1—H1B	109.5
C75—C74—C73	120.88 (16)	N2—C1—H1C	109.5
C75—C74—Cl1	119.19 (15)	H1A—C1—H1C	109.5
C73—C74—Cl1	119.90 (15)	H1B—C1—H1C	109.5
N1—C8—C9	110.34 (15)	C54—C53—C52	121.8 (2)
N1—C8—C7	100.73 (13)	C54—C53—H53	119.1
C9—C8—C7	115.74 (16)	C52—C53—H53	119.1
N1—C8—H8	109.9	C56—C57—C52	121.09 (19)
C9—C8—H8	109.9	C56—C57—H57	119.5
C7—C8—H8	109.9	C52—C57—H57	119.5
C24—C15—C16	119.59 (17)	C9—C10—C11	110.04 (18)
C24—C15—C14	132.85 (18)	C9—C10—H10A	109.7
C16—C15—C14	107.46 (15)	C11—C10—H10A	109.7
C73—C72—C71	121.41 (17)	C9—C10—H10B	109.7
C73—C72—H72	119.3	C11—C10—H10B	109.7
C71—C72—H72	119.3	H10A—C10—H10B	108.2
N2—C2—C3	108.72 (13)	C55—C56—C57	119.6 (2)
N2—C2—H2A	109.9	C55—C56—H56	120.2
C3—C2—H2A	109.9	C57—C56—H56	120.2
N2—C2—H2B	109.9	C10—C11—C12	110.47 (18)
C3—C2—H2B	109.9	C10—C11—H11A	109.6
H2A—C2—H2B	108.3	C12—C11—H11A	109.6
C51—C5—C6	124.03 (17)	C10—C11—H11B	109.6
C51—C5—C4	116.83 (16)	C12—C11—H11B	109.6
C6—C5—C4	119.11 (15)	H11A—C11—H11B	108.1
			
C8—N1—C13—C17	154.02 (14)	C21—C16—C15—C24	−2.6 (3)
C12—N1—C13—C17	−74.14 (19)	C17—C16—C15—C14	−3.0 (2)
C8—N1—C13—C14	−92.89 (16)	C21—C16—C15—C14	174.40 (16)
C12—N1—C13—C14	39.0 (2)	O2—C14—C15—C24	12.2 (3)
C8—N1—C13—C3	28.69 (17)	C13—C14—C15—C24	−176.95 (19)
C12—N1—C13—C3	160.53 (15)	O2—C14—C15—C16	−164.31 (18)
C2—N2—C6—C5	52.69 (19)	C13—C14—C15—C16	6.59 (18)
C1—N2—C6—C5	179.04 (16)	C76—C71—C72—C73	−0.2 (3)
C8—C7—C71—C72	146.51 (17)	C7—C71—C72—C73	−178.48 (16)
C3—C7—C71—C72	−92.2 (2)	C1—N2—C2—C3	161.35 (16)
C8—C7—C71—C76	−31.7 (2)	C6—N2—C2—C3	−72.88 (18)
C3—C7—C71—C76	89.6 (2)	C4—C3—C2—N2	61.91 (17)
C15—C16—C21—C20	−175.04 (17)	C7—C3—C2—N2	−173.22 (14)
C17—C16—C21—C20	2.1 (3)	C13—C3—C2—N2	−58.09 (18)
C15—C16—C21—C22	3.5 (3)	C52—C51—C5—C6	2.4 (3)
C17—C16—C21—C22	−179.40 (16)	C52—C51—C5—C4	−179.48 (18)
O1—C4—C3—C2	143.79 (16)	N2—C6—C5—C51	150.50 (18)
C5—C4—C3—C2	−37.17 (19)	N2—C6—C5—C4	−27.6 (2)
O1—C4—C3—C7	19.3 (2)	O1—C4—C5—C51	22.6 (2)
C5—C4—C3—C7	−161.68 (14)	C3—C4—C5—C51	−156.42 (16)
O1—C4—C3—C13	−95.18 (18)	O1—C4—C5—C6	−159.19 (17)
C5—C4—C3—C13	83.86 (17)	C3—C4—C5—C6	21.8 (2)
C71—C7—C3—C2	−33.4 (2)	C5—C51—C52—C57	31.6 (3)
C8—C7—C3—C2	94.06 (16)	C5—C51—C52—C53	−151.9 (2)
C71—C7—C3—C4	88.23 (18)	C16—C15—C24—C23	−0.4 (3)
C8—C7—C3—C4	−144.31 (14)	C14—C15—C24—C23	−176.51 (19)
C71—C7—C3—C13	−153.41 (14)	C72—C71—C76—C75	−0.7 (3)
C8—C7—C3—C13	−25.94 (16)	C7—C71—C76—C75	177.56 (18)
N1—C13—C3—C2	−121.20 (14)	C18—C19—C20—C21	−1.0 (3)
C17—C13—C3—C2	118.03 (16)	C16—C21—C20—C19	−0.1 (3)
C14—C13—C3—C2	2.04 (19)	C22—C21—C20—C19	−178.4 (2)
N1—C13—C3—C4	120.25 (14)	C15—C24—C23—C22	2.4 (3)
C17—C13—C3—C4	−0.5 (2)	C8—N1—C12—C11	−58.0 (2)
C14—C13—C3—C4	−116.51 (15)	C13—N1—C12—C11	172.85 (16)
N1—C13—C3—C7	−0.43 (16)	C16—C17—C18—C19	1.7 (3)
C17—C13—C3—C7	−121.19 (15)	C13—C17—C18—C19	177.35 (17)
C14—C13—C3—C7	122.81 (14)	C20—C19—C18—C17	0.2 (3)
C15—C16—C17—C18	174.47 (16)	C75—C74—C73—C72	−0.9 (3)
C21—C16—C17—C18	−2.9 (3)	Cl1—C74—C73—C72	177.12 (15)
C15—C16—C17—C13	−2.0 (2)	C71—C72—C73—C74	1.0 (3)
C21—C16—C17—C13	−179.45 (15)	C73—C74—C75—C76	0.0 (3)
N1—C13—C17—C18	−49.0 (2)	Cl1—C74—C75—C76	−177.98 (15)
C14—C13—C17—C18	−170.25 (18)	C71—C76—C75—C74	0.8 (3)
C3—C13—C17—C18	68.1 (2)	C56—C55—C54—C53	−0.9 (4)
N1—C13—C17—C16	126.93 (15)	Cl2—C55—C54—C53	178.73 (18)
C14—C13—C17—C16	5.71 (17)	N1—C8—C9—C10	−55.0 (2)
C3—C13—C17—C16	−115.96 (16)	C7—C8—C9—C10	−168.55 (17)
N1—C13—C14—O2	45.7 (2)	C24—C23—C22—C21	−1.5 (3)
C17—C13—C14—O2	163.85 (17)	C16—C21—C22—C23	−1.4 (3)
C3—C13—C14—O2	−71.0 (2)	C20—C21—C22—C23	176.9 (2)
N1—C13—C14—C15	−125.51 (15)	C55—C54—C53—C52	1.3 (3)
C17—C13—C14—C15	−7.35 (17)	C57—C52—C53—C54	−1.0 (3)
C3—C13—C14—C15	117.78 (15)	C51—C52—C53—C54	−177.8 (2)
C12—N1—C8—C9	57.7 (2)	C53—C52—C57—C56	0.2 (3)
C13—N1—C8—C9	−168.56 (15)	C51—C52—C57—C56	176.79 (18)
C12—N1—C8—C7	−179.52 (15)	C8—C9—C10—C11	55.3 (3)
C13—N1—C8—C7	−45.76 (17)	C54—C55—C56—C57	0.1 (3)
C71—C7—C8—N1	171.17 (14)	Cl2—C55—C56—C57	−179.47 (16)
C3—C7—C8—N1	43.08 (16)	C52—C57—C56—C55	0.2 (3)
C71—C7—C8—C9	−69.9 (2)	C9—C10—C11—C12	−56.0 (3)
C3—C7—C8—C9	162.04 (15)	N1—C12—C11—C10	56.2 (3)
C17—C16—C15—C24	179.99 (16)		

### Hydrogen-bond geometry (Å, º)

Cg1 is the centroid of the C52–C57 benzene ring.

**Table:**

*D*—H···*A*	*D*—H	H···*A*	*D*···*A*	*D*—H···*A*
C7—H7···O1	0.98	2.38	2.826 (2)	107
C2—H2*A*···O2	0.97	2.45	2.951 (2)	112
C75—H75···*Cg*1^i^	0.93	2.88	3.669 (2)	144

Symmetry code: (i) *x*−1/2, −*y*+1/2, *z*+1/2.
